# Anticoagulation management in intracerebral hemorrhage patients with deep vein thrombosis: insights from unsupervised machine learning and nomogram analysis

**DOI:** 10.3389/fneur.2025.1711123

**Published:** 2026-01-05

**Authors:** Chaohua Cui, Qiulian Yin, Tonghua Long, Haoye Guan, Zhenxian Lao

**Affiliations:** Life Science and Clinical Medicine Research Center, Affiliated Hospital of Youjiang Medical University for Nationalities, Baise, China

**Keywords:** anticoagulation risk assessment, deep vein thrombosis, intracerebral hemorrhage, nomogram analysis, unsupervised machine learning

## Abstract

**Introduction:**

Lower extremity deep vein thrombosis (DVT) is a frequent complication in patients with intracerebral hemorrhage (ICH), increasing the risk of adverse outcomes and mortality. However, standard anticoagulation therapy can lead to hematoma expansion, highlighting the need for reliable and practical risk assessment tools. While unsupervised machine learning has shown promise in patient stratification, its clinical applicability is limited. This study integrates unsupervised machine learning with nomogram analysis to identify risk factors and establish a clinically actionable risk assessment tool.

**Methods:**

The study was conducted in two phases. In the retrospective exploratory phase, 191 ICH patients receiving anticoagulation were grouped using K-means and hierarchical clustering. Incidence rates of DVT and adverse events were analyzed to identify key risk factors influencing anticoagulant safety. A nomogram was then constructed to quantify adverse event risk. In the prospective validation phase, 291 patients were stratified into high- and low-risk groups based on nomogram scores. VTE and adverse event rates were compared between groups, with multivariate regression and subgroup analyses performed.

**Results:**

Key risk factors identified included admission mRS, GCS, and ADL scores, admission and discharge ICH volume, and admission albumin level. In the validation cohort, the low-risk group had significantly lower VTE (16.9% vs. 65.1%, *p* < 0.001) and adverse event rates (13.4% vs. 46.3%, *p* < 0.001) than the high-risk group. Multivariate regression confirmed a significant inverse association between low-risk classification and occurrence of VTE and adverse events.

**Conclusion:**

This study demonstrates that unsupervised machine learning, combined with a nomogram, can effectively stratify risk in ICH patients receiving anticoagulation. The risk assessment tool reliably identifies patients at lower risk of adverse outcomes, supporting safer and more individualized clinical decision-making.

## Introduction

Deep vein thrombosis(DVT) is a common complication among patients with intracerebral hemorrhage(ICH), associated with poor prognosis, increased mortality, and reduced quality of life (1–3). Anticoagulant therapy remains the standard of care for deep vein thrombosis (DVT) prevention and management. However, its use in ICH patients carries a risk of hematoma expansion, hemorrhage recurrence, and exacerbation of their clinical condition (3, 4). This has generated considerable controversy regarding whether anticoagulants should be administered to such patients (4–7). Reflecting this uncertainty, clinical practice shows limited adoption: a Chinese study revealed only about 5% of patients received anticoagulant therapy, and parallel research in the United States reported less than 20% use (1, 8). The competing priorities of preventing DVT and avoiding further cerebral bleeding create considerable uncertainty, making this an area with significant therapeutic challenges and unmet clinical needs.

Unsupervised machine learning methods have emerged as powerful tools to uncover hidden patterns and interactions among clinical features without the need for labeled outcomes (9–14) These approaches can be instrumental in identifying previously unrecognized factors influencing disease course and treatment response. Findings from these studies suggested that unsupervised machine learning could improve safety profiles and optimize treatment strategies by illuminating nuanced associations and potential risks that traditional analytical techniques might overlook. Accordingly, clustering and other tools from unsupervised learning may be leveraged to discover safe and effective anticoagulant regimens for ICH patients with DVT (12–14). This study employed unsupervised machine learning methods to effectively classify the data and identify several key risk factors affecting the safety of anticoagulant use.

Despite their promise, predictions and stratifications derived from unsupervised machine learning methods are not readily transferable to the clinical setting. The lack of an intuitive interface and the complexity of algorithmic outputs can hinder real-world application by clinicians (13, 15–17). Nomograms, on the other hand, offer a robust and accessible tool for personalized risk prediction, transforming complex statistical results into visual aids that facilitate bedside decision-making (18, 19). By integrating clinical variables and probabilistic outcomes, nomograms bridge the gap between computational insights and clinical usability, empowering practitioners to make more informed, patient-specific therapeutic choices. In the validation cohort of this study, the nomogram was found to provide effective risk assessment for anticoagulant use in patients with intracerebral hemorrhage.

In light of these considerations, our present study was designed to explore anticoagulant therapy strategies for patients with ICH complicated by DVT, leveraging unsupervised machine learning in conjunction with nomogram development. We aimed to elucidate patterns in therapeutic safety and efficacy, construct predictive visual models for guiding treatment decisions, and rigorously validate the utility and risk profile of the proposed strategies. The integration of data-driven insights with user-friendly clinical tools may offer a novel pathway toward safer and more effective management in this challenging patient population.

## Methods

### Cohort 1: exploratory cohort

#### Cohort characteristics

This study was a retrospective observational cohort, including consecutively admitted intracerebral hemorrhage (ICH) patients who received anticoagulant therapy. These patients were hospitalized in the Department of Neurosurgery and Rehabilitation at the Affiliated Hospital of Youjiang Medical University for Nationalities from January 1, 2018 to December 31, 2020. The inclusion criteria comprised: (1) age ≥18 years; (2) primary diagnosis of spontaneous ICH confirmed by neuroimaging; (3) complete medical records; and (4) receipt of anticoagulant medication during admission. Patients were excluded if they had: (1) traumatic ICH, acute spontaneous lobar cerebral hemorrhagesor and secondary hemorrhage from underlying vascular malformations, tumors, or hemorrhagic transformation of ischemic stroke; (2) pre-existing coagulopathy; (3) pre-existing intracerebral hemorrhage or gastrointestinal bleeding; (4) pre-existing deep vein thrombosis or pulmonary embolism; (5) incomplete baseline or follow-up data; or (6) contraindications to anticoagulation.

The study protocol was approved by the institutional ethics committee, and all procedures were conducted in accordance with the Declaration of Helsinki. Patient consent was obtained where required by the institutional review board.

#### Data collection

Relevant clinical data were systematically extracted from the electronic medical records. Demographic information included age, sex, and ethnicity. Baseline medical history encompassed hypertension, diabetes, atrial fibrillation, prior stroke, and other comorbidities. Medication history was recorded, particularly focusing on prior antithrombotic or anticoagulant use. In-hospital complications, including infections and secondary ischemic or hemorrhagic events, were documented. Treatment variables included the type, dosage, and duration of anticoagulation, as well as adjunctive therapies. Neurological function was evaluated using standardized scales: National Institutes of Health Stroke Scale (NIHSS), modified Rankin Scale (mRS), Glasgow Coma Scale (GCS), and Activities of Daily Living (ADL) scores, both at admission and discharge. Hematoma volume and changes were calculated based on admission and discharge neuroimaging. Laboratory data included D-dimer, platelet count, international normalized ratio (INR), and other relevant coagulation markers. The primary effectiveness endpoint was the occurrence of lower extremity DVT, confirmed by Doppler ultrasonography or computed tomography venography during hospitalization. Safety outcomes included ICH recurrence, hematoma expansion, gastrointestinal bleeding, and all-cause mortality.

#### Unsupervised machine learning exploration

Unsupervised machine learning methods were employed to stratify risk factors among ICH patients treated with anticoagulants. During the analysis, Python 3.8 was used for machine learning computations. Data were first standardized using the StandardScaler module from the sklearn library. The K-means algorithm and hierarchical clustering analyses were performed on the aggregated dataset, incorporating demographic, clinical, laboratory, and treatment variables. Scatterplots clearly demonstrated the group characteristics identified by K-means, while heatmaps distinctly illustrated the features of groups derived from clustering. We compared the incidence rates of safety and efficacy endpoints among the groups identified by both classification methods. Further analysis of these factors within groups was performed using chi-square tests and t-tests. Risk factors that differed between groups with significant event differences may influence the safety of anticoagulant therapy.

#### Nomogram construction

Risk factors identified through unsupervised clustering were subsequently incorporated into a multivariate logistic regression model to construct a predictive nomogram. This nomogram was designed to estimate the probability of adverse events during anticoagulant therapy, specifically focusing on ICH recurrence, hematoma expansion, gastrointestinal bleeding, and mortality. Variables selected for the final model were those demonstrating significant intergroup differences and clinical relevance, as determined by clustering and risk factor analysis. The predictive performance of the nomogram was evaluated using discrimination and calibration metrics.

### Cohort 2: validation cohort

#### Cohort characteristics

This cohort was a prospective observational study, enrolling patients with intracerebral hemorrhage who received anticoagulant therapy consecutively. Patients were recruited from the Department of Neurosurgery and the Department of Rehabilitation at the Affiliated Hospital of Youjiang Medical University for Nationalities from July 1, 2020, to December 31, 2023, with follow-up continuing until December 31, 2024. The inclusion and exclusion criteria were consistent with those of the exploratory cohort. The inclusion and exclusion criteria were identical to those applied in the initial cohort. For each patient, the risk probability of anticoagulant-related adverse events was calculated using the nomogram constructed from Cohort 1. Patients with a predicted adverse event probability greater than 0.6 were classified into the high risk group, whereas those with a probability less than 0.3 were classified into the low risk group.

#### Data collection and outcome events

Demographic data, medical history, prior medication use, in-hospital complications, treatment details, clinical scores, imaging, and laboratory results were collected in accordance with the protocol of Cohort 1. The primary effectiveness outcome was defined as the occurrence of deep vein thrombosis (DVT) during the follow-up period, confirmed by Doppler ultrasound or CT venography according to standard diagnostic criteria. The primary safety outcomes was adverse events included the incidence of bleeding events and all-cause mortality, ascertained through clinical records and follow-up interviews. Bleeding events were categorized based on the International Society on Thrombosis and Haemostasis (ISTH) criteria. Anticoagulant medications tracked in this study included low molecular weight heparin, direct oral anticoagulants (DOACs), and warfarin, with specific dosing and duration recorded for each patient. The follow-up duration for all outcomes was set at one year from the date of cohort entry. During the one-year follow-up, all patients underwent routine outcome event screenings every 3 months, while patients showing relevant symptoms outside of follow-up periods were immediately screened, with the decision to continue follow-up or withdraw from the study based on their clinical condition.

### Statistical analysis

Statistical analyses were performed using SPSS 23.0 for Windows. For baseline data comparisons, continuous variables were analyzed using t-tests or non-parametric tests based on their distribution; categorical and ordinal variables were analyzed using chi-square tests. The incidence rates of VTE, bleeding events, and death among different groups were also compared using chi-square tests.

Subsequently, univariate and multivariate logistic regression analyses were conducted to identify risk factors for both efficacy and safety outcomes. Variables for multivariate analysis were selected based on clinical relevance and results from univariate analyses. The relationship between nomogram-based group assignments and clinical outcomes, particularly safety endpoints, was evaluated. Subgroups were classified according to age, gender, and disease severity. The gender subgroups consisted of male and female groups. The age subgroups were divided into elder (patients aged 60 years or older) and younger groups (patients younger than 60 years). Disease severity subgroups were classified as mild (admission mRS score less than 4) and severe (admission mRS score of 4 or higher).

## Results

### Cohort 1: exploratory cohort

**Figure 1:**
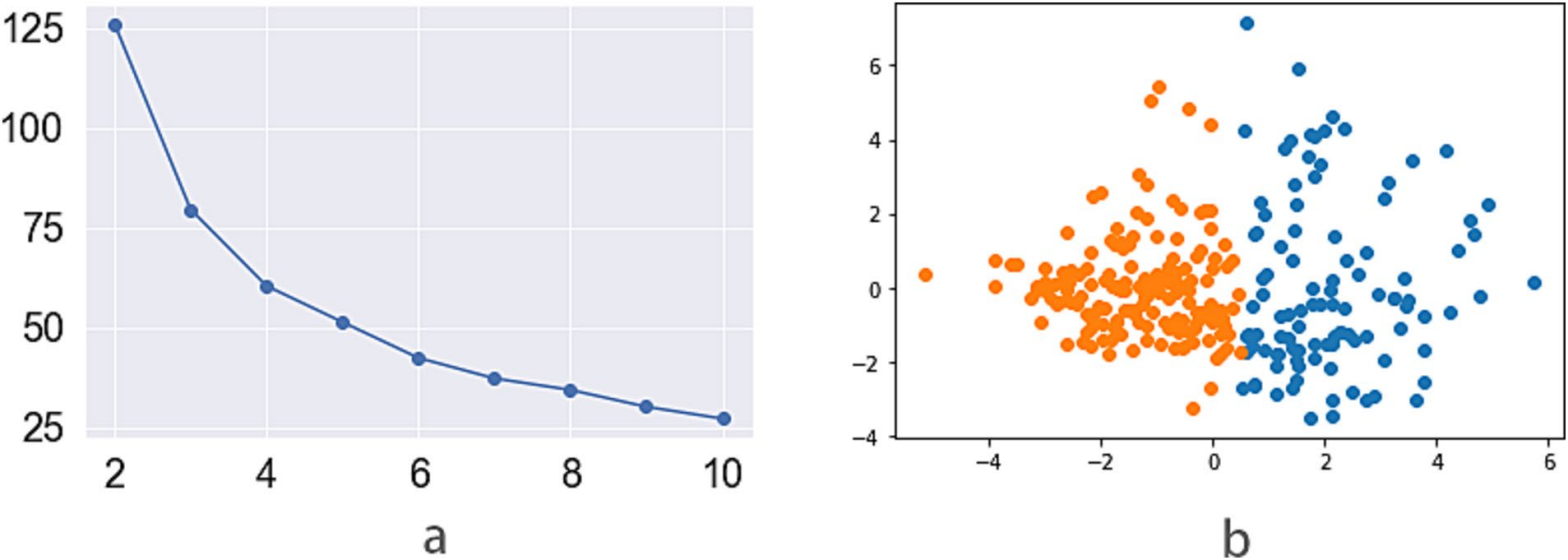
**(a)** The line chart of silhouette score for k-means methods (vertical and horizontal axes were cluster number and the silhouette score, the two groups had a highest silhouette score); **(b)** The scatter plots of 2 groups by k-means (km1 group, orange; km2 group, blue).

**Figure 2:**
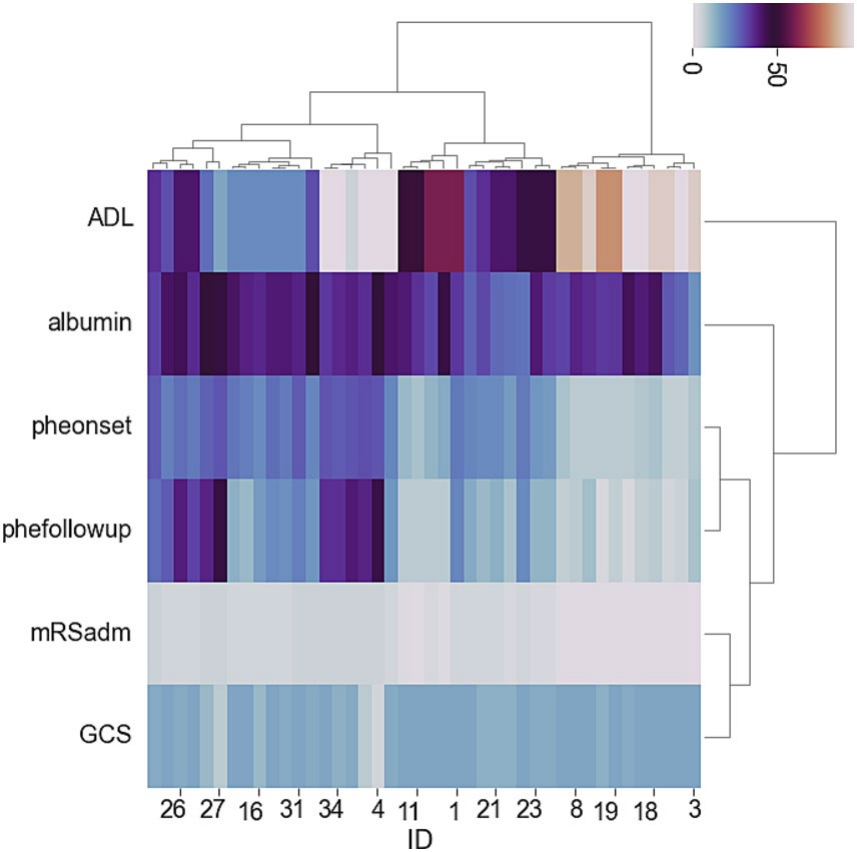
Heat-map showing hierarchical clustering methods for adverse events and associated risk factors in ICH patients.

The exploratory cohort included 191 patients, of whom 82 were female (42.93%), with a mean age of 72.45 ± 11.357 years. The silhouette coefficient from the K-means method indicated that dividing the data into two groups (K1 and K2) was optimal (Figure 1a), and the scatter plot further demonstrated a clear distinction between these groups with satisfactory clustering results (Figure 1b). The heatmap from the clustering method (Figure 2) showed that two groups (H1 and H2) were most suitable for hierarchical clustering.

Comparison of efficacy and safety outcome rates between the groups revealed no significant difference in the incidence of VTE between K1 and K2 (*p* = 0.069) or between H1 and H2 (*p* = 0.102). In terms of mortality, K1 (11.0%) was lower than K2 (45.8%) (*p* < 0.001), and H1 (15.7%) was lower than H2 (45.1%) (*p* < 0.001). Regarding bleeding events, K1 (12.3%) was lower than K2 (25.7%) (*p* = 0.028), and H1 (14.4%) was lower than H2 (22.3%) (*p* = 0.043). Therefore, both unsupervised clustering methods effectively distinguished the safety profiles of patients with intracerebral hemorrhage receiving anticoagulation, while no significant difference was observed in the efficacy of VTE treatment and prevention.

Further comparison of risk factors between K1 and K2 revealed that patients in K1 had shorter hospital stays, lower NIHSS and mRS scores at admission, higher GCS and ADL scores at admission, smaller hematoma volumes at admission, and lower D-dimer levels at admission. For H1 compared to H2 patients, besides the similarities in admission scores seen with K1 and K2, H1 also had smaller hematoma volumes at discharge, a notable reduction in bleeding during hospitalization (defined as a reduction of more than 5 mL or 30% from admission to discharge), and higher serum albumin levels at admission.

**Figure 3:**
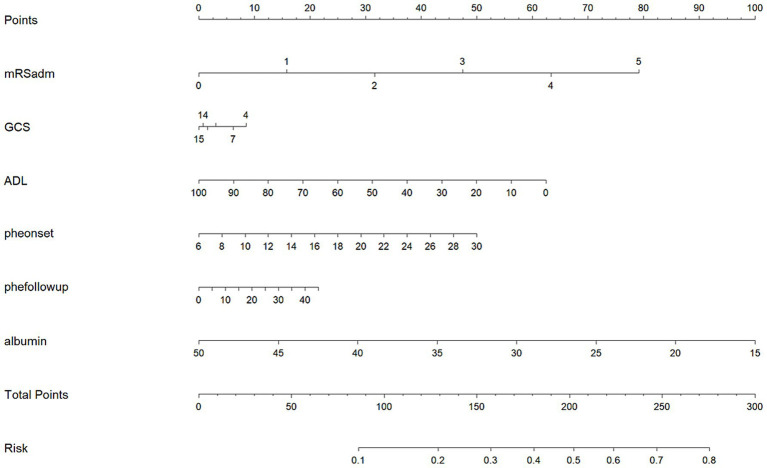
Nomogram for assessing the risk of adverse events in patients with intracerebral hemorrhage treated with anticoagulants (mRSadm, mRS score at admission; pheonset, intracerebral hemorrhage volume at admission; phefollowup, intracerebral hemorrhage volume 2–3 weeks after onset).

Incorporating these risk factors into a logistic regression model for safety outcomes showed that six variables remained correlated with adverse events: admission mRS score, GCS score, and ADL score; hematoma volume at admission and discharge; and serum albumin level at admission. These variables were used as risk factors to develop a nomogram for adverse outcomes using logistic regression (Figure 3). The nomogram indicated that when the risk score was below 90 points, the probability of adverse events from anticoagulant use was less than 0.1 (10%), while when the score exceeded 280 points, the probability was greater than 0.8 (80%).

### Cohort 2: validation cohort

#### Cohort characteristics

A total of 312 participants were initially recruited for this cohort. Of these, 11 patients withdrew or were lost to follow-up, 3 were excluded due to exclusion criteria, and 7 died within one month after disease onset. Ultimately, the validation cohort comprised 291 eligible patients, with 149 patients in the low-risk group and 142 in the high-risk group.

The mean age of the validation cohort was 62.94 ± 13.183 years, and there were 965 female patients, accounting for 33.0% of the cohort. When comparing baseline data, the high-risk group exhibited a higher proportion of patients with cognitive impairment (*p* = 0.035), a greater proportion with a history of stroke (*p* = 0.035), longer hospital stays (*p* = 0.047), higher admission NIHSS (*p* = 0.001) and mRS scores (*p* = 0.004), lower admission GCS (*p* < 0.001) and ADL scores (*p* = 0.004), and a greater volume of intracerebral hemorrhage at discharge (*p* < 0.001). No significant differences were found in other baseline characteristics between the groups (Table 1).

**Table 1:** Baseline characteristic data.

Risk factor	All patients (*N* = 291)	Low risk group (*N* = 142)	High riskgroup (*N* = 149)	*P*
Baseline characteristic				
Age, years	62.94 (13.183)	62.25 (12.050)	63.60 (14.188)	0.386
Female, %	96 (33.0)	47 (31.1)	49 (32.9)	0.620
mRS score	4 (2–4)	3 (2–4)	4 (2–5)	**0.004**
NIHSS score	10 (4–10)	8 (3–15)	12 (5–23)	**0.001**
GCS score	14 (13–15)	14 (14–15)	14 (12–15)	**<0.001**
ADL score	40 (20–70)	45 (25–70)	30 (10–60)	**0.004**
SBP, mmHg	148.90 (28.080)	147.66 (26.111)	150.07 (29.877)	0.465
DBP, mmHg	86.95 (16.821)	86.12 (15.167)	87.74 (18.274)	0.412
Day of bed, day	14.35 (3.367)	13.23 (4.249)	15.41 (2.349)	**0.047**
Adm hematoma, ml	18.32 (7.391)	17.49 (6.471)	19.12 (8.115)	0.058
Dis hematoma, ml	17.08 (12.273)	13.81 (7.712)	20.19 (14.781)	**<0.001**
Hypertension, %	174 (59.8)	78 (54.9)	96 (64.4)	0.120
Diabetes Mellitus, %	63 (21.6)	29 (20.4)	34 (22.8)	0.670
History of stroke, %	65 (22.3)	24 (16.9)	41 (27.5)	**0.035**
Pneumonia, %	111 (38.1)	52 (36.6)	59 (39.6)	0.630
Epilepsy, %	14 (4.8)	5 (3.5)	9 (6.0)	0.414
Anaemia, %	71 (24.4)	36 (25.4)	35 (23.5)	0.785
Ion disturbance, %	143 (49.1)	70 (49.3)	73 (49.0)	0.959
Renal insuf, %	12 (4.1)	5 (3.5)	7 (4.7)	0.770
Atherosclerosis, %	96 (33.0)	53 (37.3)	43 (28.9)	0.136
Cognitive diso, %	12 (4.1)	2 (1.4)	10 (6.7)	**0.035**
Dehydrant drug, %	198 (68.0)	93 (65.5)	105 (70.5)	0.381
Antiinfe drug, %	119 (40.9)	54 (38.0)	65 (43.6)	0.343
Laboratory data at admission				
Platelet, mmol/l	198.07 (71.258)	197.70 (60.800)	198.42 (80.171)	0.932
INR	1.049 (0.204)	1.034 (0.129)	1.065 (0.256)	0.197
D-Dimer, mmol/l	1.24 (1.732)	0.88 (1.938)	1.52 (1.681)	0.097
ALT, mmol/l	23.30 (16.037)	24.32 (17.193)	22.34 (14.846)	0.293
AST, mmol/l	26.85 (15.443)	27.85 (14.759)	25.89 (16.059)	0.279
Creatinine, mmol/l	82.46 (37.648)	81.10 (33.094)	83.75 (41.598)	0.547
Glucose, mmol/l	8.382 (4.647)	7.68 (3.535)	9.06 (5.905)	0.313
Triglyceride, mmol/l	1.54 (1.076)	1.72 (1.333)	1.36 (0.712)	**0.004**
TC, mmol/l	4.29 (1.004)	4.35 (0.996)	4.24 (1.007)	0.362
HDL-C, mmol/l	1.27 (0.405)	1.24 (0.363)	1.29 (0.443)	0.961
LDL-C, mmol/l	2.51 (0.811)	2.53 (0.843)	2.48 (0.781)	0.595

P* was calculated using ANOVA, Chi-square test, or Mann–Whitney U test as appropriate between low risk group and high risk group. NIHSS, National Institute of Health Stroke Scale at admission; mRS, Modified Rankin Scale at admission; GCS, Glasgow Coma Scale at admission; ADL, activity of daily living at admission, SBP, systolic blood pressure at admission; DBP, diastolic blood pressure at admission; Adm hematoma, hematoma value at admission; Dis hematoma, hematoma value at discharge; Renal Insuf, renal insufficiency; Cognitive dis, cognitive disorder; Antiinfe drug, Antiinfection drug; INR, international normalized ratio; ALT, alanine aminotransferase (formerly known as glutamic-pyruvic transaminase); AST, aspartate aminotransferase (formerly known as glutamic-oxalacetic transaminase); TC, total cholesterol, HDL-C, high-density lipoprotein cholesterol; LDL-C, low-density lipoprotein cholesterol. Bold font indicates *P* < 0.05, suggesting that the difference is statistically significant.

#### Outcome events

##### Incidence of VTE

The results showed that the incidence of VTE was significantly lower in the low-risk group (16.9%) compared to the high-risk group (65.1%) (*p* < 0.001) (Table 2). Multivariate logistic regression analysis revealed that the low-risk group (OR = 0.028, *p* < 0.001) and using antihypertensive drugs (OR = 0.078, *p* = 0.015) was significantly negatively associated with VTE occurrence. In contrast, female sex (OR = 7.104, *p* < 0.001), older age (OR = 1.053, *p* = 0.009), prolonged bed rest (OR = 1.422, *p* < 0.001), higher NIHSS score on admission (OR = 1.211, *p* = 0.001) and elevated D-dimer level at admission (OR = 1.793, *p* ≤ 0.001) were all positively associated with VTE (Table 3). Subgroup analysis demonstrated that the negative association between the low-risk group and VTE incidence persisted across subgroups based on age, sex, and disease severity (Supplementary Figure 1).

**Table 2:** The outcome of validation cohort in difference group (%).

Group	Low risk group (*N* = 142)	High risk group (*N* = 149)	*P*
VTE (%)	24 (16.9)	97 (65.1)	<0.001
Adverse events (%)	19 (13.4)	69 (46.3)	<0.001

P* was calculated by Chi-square test. VTE, venous thromboembolism.

**Table 3:** Multivariate logistic regression for outcome.

Risk factor	OR (95%CI)	P*
VTE		
Low-risk group	0.028 (0.007–0.111)	<0.001
Antihypertensive drugs	0.078 (0.010–0.609)	0.015
Prolonged bed rest	1.422 (1.197–1.689)	<0.001
Female	7.104 (2.480–12.354)	<0.001
Older	1.053 (1.013–1.095)	0.009
Higher NIHSS score at admission	1.211 (1.086–1.350)	0.001
Higher D-dimer at admission	1.793 (1.267–2.537)	0.001
Adverse events		
Low-risk group	0.032 (0.003–0.315)	0.003
Male	0.018 (0.002–0.179)	<0.001
Higher NIHSS Score at Admission	1.936 (1.371–2.735)	<0.001
Larger hematoma volume at discharge	1.542 (1.278–1.862)	<0.001
Higher CRP level at admission	1.140 (1.063–1.223)	<0.001
Higher D-dimer at admission	2.186 (1.665–3.097)	<0.001

P* was calculated by multivariate logistic regression. VTE, venous thromboembolism; NIHSS, National Institute of Health Stroke Scale at admission; CRP, C-reactive protein.

##### Adverse events

The results showed that the incidence of adverse events was significantly lower in the low-risk group (13.4%) compared to the high-risk group (46.3%) (*p* < 0.001) (see Table 2).

Multivariate logistic regression analysis indicated that the low-risk group (OR = 0.032, *p* = 0.003) and male (OR = 0.018, *p* < 0.001) was significantly negatively associated with the occurrence of adverse events. In contrast, higher NIHSS score at admission (OR = 1.936, *p* < 0.001), larger hematoma volume at discharge (OR = 1.542, *p* < 0.001), higher CRP level at admission (OR = 1.140, *p* = 0.009), and elevated D-dimer level at admission (OR = 2.186, *p* < 0.001) were all positively associated with the occurrence of adverse events (Table 3).

Subgroup analysis showed that the significant negative association between the low-risk group and adverse event incidence was maintained in subgroups with older age, female sex, and more severe conditions, but was not observed in the subgroup with milder disease severity (Supplementary Figure 2).

## Discussion

Using K-means and cluster analysis, we identified potential risk factors for anticoagulant use in the exploratory cohort, including admission mRS, GCS, and ADL scores; intracerebral hemorrhage volume at both admission and discharge; and admission albumin levels. A nomogram was constructed based on these six variables. In the validation cohort, this nomogram was applied to assess the safety risks of anticoagulant therapy in patients with intracerebral hemorrhage, categorizing them into high- and low-risk groups. The low-risk group experienced lower rates of VTE, mortality, intracerebral hemorrhage recurrence, and gastrointestinal bleeding compared to the high-risk group. Importantly, the association between the low-risk group and reduced incidence of VTE and adverse events after anticoagulant use remained significant across different age and gender subgroups. This correlation persisted in the severe subgroup and was specifically linked to lower VTE incidence in the mild subgroup.

Unsupervised machine learning enables the classification and exploration of unknown risk factors. In our previous research, we effectively employed methods such as K-means and hierarchical clustering to identify factors influencing elevated transaminases in ischemic stroke patients treated with statins, as well as gastrointestinal bleeding in those treated with antiplatelet agents (12, 13). We also explored prognostic factors in ischemic stroke patients undergoing transcranial magnetic stimulation therapy (14). In the present study, unsupervised machine learning similarly facilitated the identification of factors associated with adverse outcomes in intracerebral hemorrhage patients receiving anticoagulants for VTE prevention.

While previous studies have identified risk factors, the interpretability and clinical applicability of machine learning model coefficients remain limited (12–17). In contrast, nomograms provide a more accessible and practical tool for clinical risk and prognostic assessment. Previously, we utilized nomograms to evaluate the incidence of VTE (18). In this study, we went beyond merely identifying risk factors by converting them into a practical risk assessment tool using a nomogram, thereby enabling our findings to be directly translated into clinical practice.

Two unsupervised machine learning methods identified different risk factors associated with adverse outcomes. Among the risk factors included in the nomogram, the mRS score at admission and the volume of intracerebral hemorrhage at both admission and discharge were consistent with the features used in our previous model for intracerebral hemorrhage recurrence, and were similarly recognized as risk factors in other studies (20–22). Patients with higher GCS scores tend to be more alert, experience shorter periods of complete bed rest, are less likely to develop complications such as infections, and generally have milder conditions, which lowers the likelihood of adverse events. Likewise, patients with higher ADL scores, due to less time spent bedridden and greater ability for self-care and mobility, typically have milder illnesses and face a reduced risk of recurrent hemorrhage and death. Furthermore, the albumin level at admission serves as an overall indicator of the patient’s physical condition. Patients with very low albumin levels are often more severely ill, bedridden for longer durations, or suffer from wasting conditions such as dysphagia. These individuals have lower immunity, are more susceptible to complications like infections, and are therefore at a higher risk for adverse outcomes, including death.

The proportion of patients experiencing gastrointestinal bleeding could experience worsened conditions or even death. Moreover, most patients with gastrointestinal bleeding experience issues with medication adherence, which can affect the efficacy of the drug. Therefore, we have included gastrointestinal bleeding as a safety event for evaluation. However, the analysis of risk factors for adverse events in the validation cohort differed from those in the unsupervised outcome cohort. Apart from similar NIHSS scores and hematoma volume, female gender emerged as a risk factor for adverse events, which is consistent with our previous findings and other related studies indicating poorer outcomes in women with cerebrovascular disease (20, 22). Longer bed rest duration, similar to lower ADL and GCS scores mentioned above, was associated with more severe illness and an increased likelihood of complications. Elevated CRP levels, which indicate infectious or inflammatory responses and worsen the condition, were also reasonable risk factors (23). The main reason for the differences from the unsupervised cohort may be the distinct cohort characteristics; more importantly, the inflammation cohort was stratified according to risk scores from the nomogram, and the inclusion of grouping factors likely diminished the impact of some risk factors identified in the unsupervised cohort during correlation analysis. Similar findings were observed for the risk factors associated with VTE occurrence. Elderly patients generally present with poorer physical conditions, more comorbidities, and are more likely to be bedridden, while hypertensive patients with intracerebral hemorrhage are also more susceptible to vascular lesions that can trigger VTE. Although grouping was associated with both VTE occurrence and adverse events in most subgroups, this association was not significant in the subgroup with milder illness, likely due to the lower incidence of adverse events, making this outcome reasonable.

The super-aged patients (age>85 years) have a higher risk of intracerebral hemorrhage. In our validation cohort, there are 9 super-aged patients. Further analysis of this subgroup revealed that super-aged patients using anticoagulants had a higher recurrence rate of intracerebral hemorrhage compared to other patients (*p* = 0.012). However, there were no significant differences in mortality, incidence, and recurrence rates of VTE among patients. This may be due to the small sample size of this subgroup, which affected the statistical significance of the results. Nevertheless, these results suggest that anticoagulants should be used with more caution in super-aged patients. The intracerebral hemorrhage caused by the use of anticoagulants is related to the location of bleeding, with more occurrences in the brain lobes. To avoid safety concerns related to the use of anticoagulants in such patients, we did not include these patients in our study (24). Intraventricular hemorrhage is a significant risk factor for the prognosis of intracerebral hemorrhage, greatly influencing the recurrence of bleeding and the mortality risk in affected patients. Due to the severe condition of these patients and the lower likelihood of anticoagulant use, only 7 such cases were included in our cohort. However, no significant differences were found in the incidence of adverse events and outcome events between this subgroup and the others, possibly due to the limited number of patients (25).

This study has several limitations. Firstly, as a cohort study, it can only establish associations rather than causal relationships. Secondly, because the proportion of intracerebral hemorrhage patients receiving anticoagulant therapy is relatively low, some degree of selection bias may exist. Finally, our research focused on patients deemed suitable for anticoagulant therapy. However, most individuals in the high-risk group presented with more severe and complex conditions, which not only increased their risk of deep vein thrombosis (DVT) but also led to a poorer prognosis once DVT developed. As such, the prevention and management of DVT in this subgroup remains a significant clinical challenge. For these patients, we have identified that treatments such as statins and computer-based medium-frequency stimulation are effective, and future studies will further validate and report these findings.

## Conclusion

In summary, this research utilized unsupervised machine learning to identify risk factors affecting adverse outcomes when using anticoagulants for the prevention or treatment of DVT in patients with intracerebral hemorrhage, and developed a risk assessment nomogram based on these factors. Validation in an independent cohort confirmed that patients classified as low-risk by the nomogram had a significantly lower probability of adverse events, and lower risk scores were significantly associated with fewer adverse outcomes among patients and across most subgroups. For high-risk patients identified as using anticoagulant drugs, there is currently a lack of safe and effective treatment options. Further exploration of alternative treatment options suitable for these patients is a needed research direction in clinical practice.

## Data Availability

The raw data supporting the conclusions of this article will be made available by the authors, without undue reservation.
