# Supplementary material for: Anticoagulation management in intracerebral hemorrhage patients with deep vein thrombosis: insights from unsupervised machine learning and nomogram analysis
Source: Front Neurol. 2026 Jan 5;16:1711123. doi: 10.3389/fneur.2025.1711123 (PMC12812592; doi:10.3389/fneur.2025.1711123)
Supplement: Supplementary file 1 [file Table_1.docx]

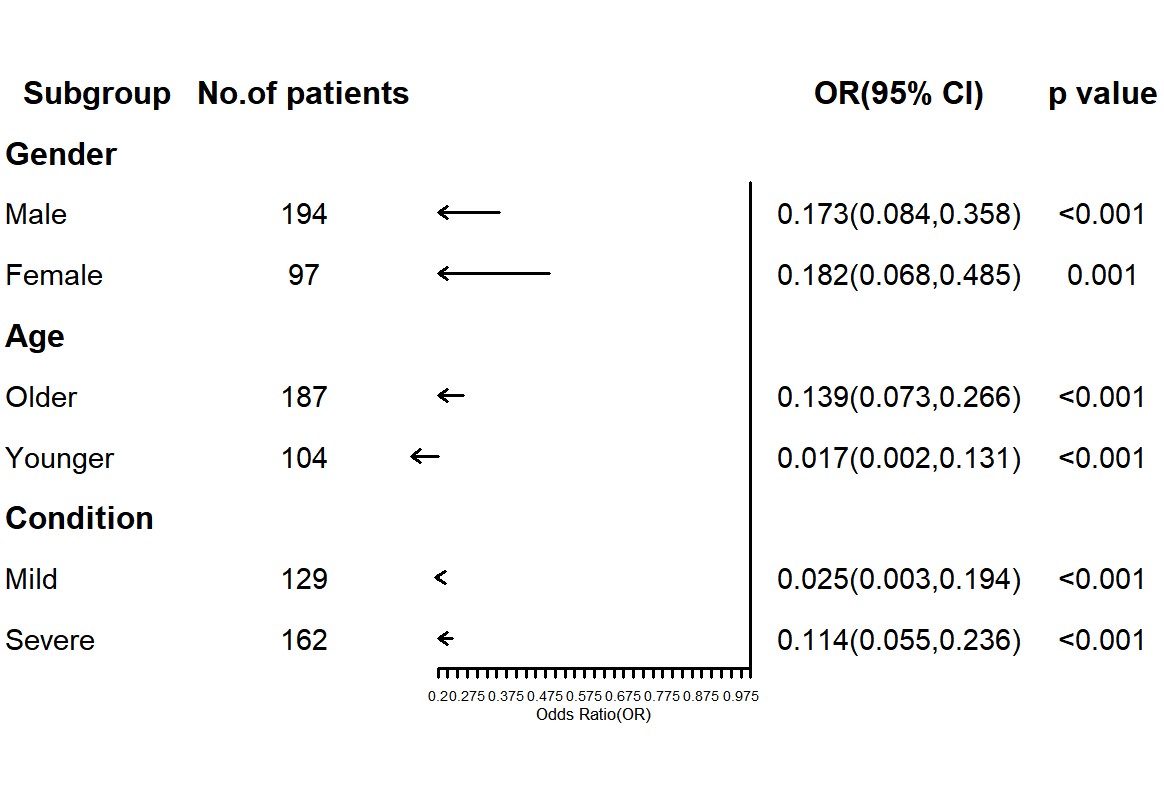


Supplementary_Figure 1. Forest plot displaying subgroup analysis of the relationship between risk group and the incidence of VTE in patients with ICH.


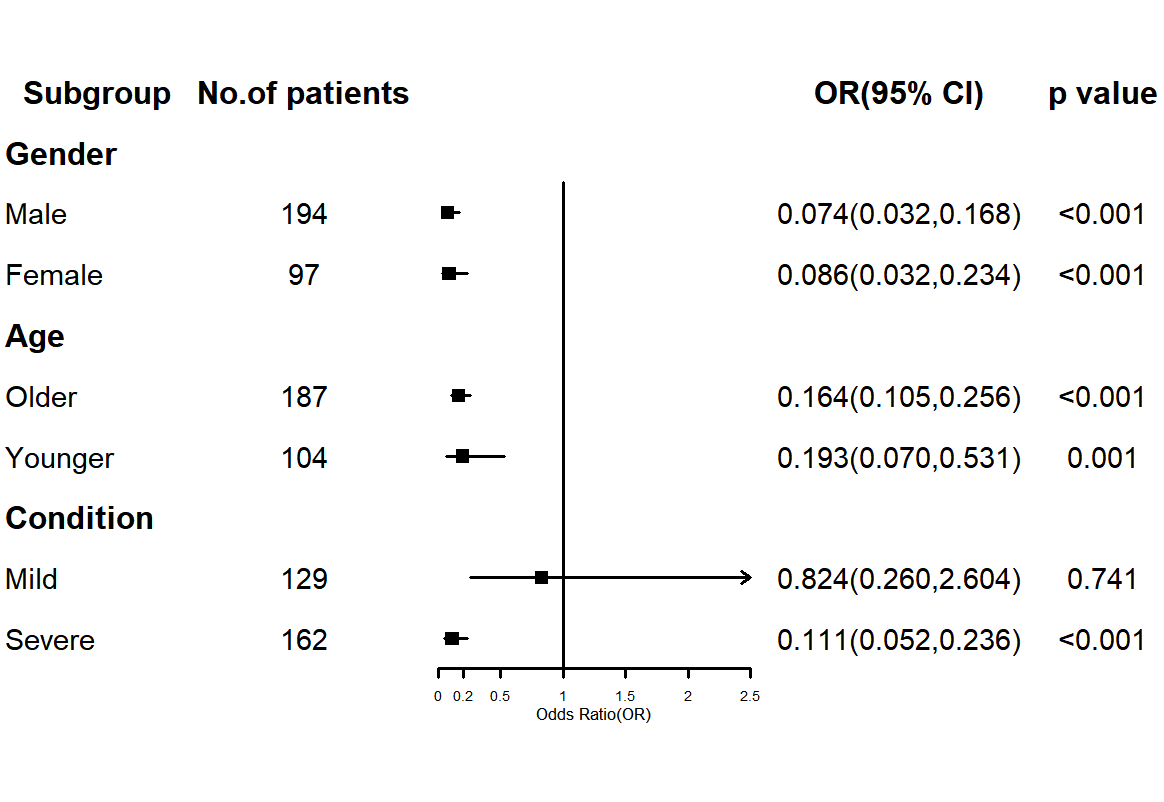


Supplementary_Figure 1. Forest plot displaying subgroup analysis of the relationship between risk group and the incidence of adverse events in patients with ICH.
